# Letter to Editor regarding ‘Variants in the interferon regulatory factor 5 gene confer genetic risk for systemic lupus erythematosus in a Han Chinese population’

**DOI:** 10.1080/07853890.2026.2635876

**Published:** 2026-03-02

**Authors:** Aditya K. Panda, Sharda Sharma

**Affiliations:** ^a^ Department of Biotechnology, ImmGen EvSys Lab, BT-113, Berhampur University, Berhampur, India; ^b^ Centre of Excellence on Bioprospecting of Ethno-pharmaceuticals of Southern Odisha (CoE-BESO), Berhampur University, Berhampur, India

**Keywords:** IRF5, systemic lupus erythematosus, gene polymorphism, population

Dear Editor

We read with great interest the recent article by Xu et al. entitled ‘Variants in the interferon regulatory factor 5 gene confer genetic risk for systemic lupus erythematosus in a Han Chinese population’ [1]. The authors address an important topic, given the established relevance of IRF5 in the pathogenesis of SLE. The authors reported an association between two functional variants (rs10954213 and rs2004640) and SLE susceptibility in the Chinese population. Although this study contributes useful data from a Northern Han Chinese cohort, several methodological and interpretational issues merit clarification.

First, regarding Hardy–Weinberg equilibrium (HWE), the control group *p* values for rs10954213 and rs2004640 were 0.093 and 0.081, respectively. These values are close to the conventional *α* threshold of 0.05 and may be considered as borderline. Although technically non-significant, such near-threshold *p* values warrant cautious interpretation rather than categorical statements that the controls are ‘genetically representative.’ Borderline deviations can reflect genotyping errors, population stratification or sampling fluctuations, especially in small sample sizes [2]. Additional quality control measures, such as duplicate genotyping, call-rate reporting or Hardy–Weinberg exact tests [3], would strengthen the confidence in the findings.

Second, the genotyping procedure requires further clarification. The authors employed PCR with sequence-specific primers (PCR-SSPs) to genotype rs10954213 and rs2004640, using 34 amplification cycles. While PCR-SSP is widely used and cost-effective, higher cycle numbers may increase the risk of non-specific amplification, allele misclassification and false-positive results, particularly in assays that rely on endpoint gel electrophoresis rather than real-time detection of PCR products. Importantly, the manuscript does not report any independent validation strategy, such as repeat genotyping of a subset of samples, blinded duplicate analyses or Sanger sequencing confirmation. In genetic association studies, it is generally recommended that a proportion (5–10%) of randomly selected samples undergo sequencing-based validation to ensure genotyping accuracy and reproducibility [4]. The absence of such quality control measures undermines confidence in the robustness of the reported genotype distributions [5], especially given the borderline HWE *p* values observed in the control group.

Third, the statistical correction strategy was inconsistent. In Table 4, Bonferroni-adjusted *p* values are presented, whereas in the methods section, the authors state that the Benjamini–Hochberg false discovery rate (FDR) approach was applied. This discrepancy creates ambiguity about the correction method governing the inference. Moreover, multiple genetic models (allelic, dominant, recessive, heterozygous and homozygous) were tested at each SNP, thereby increasing the family-wise error rate. A clear specification of the primary genetic model or a uniform multiple-testing strategy would improve analytical rigour.

In summary, while this study adds region-specific data on IRF5 polymorphisms in SLE, clarification regarding HWE interpretation, multiple testing correction and methodological issues would enhance the robustness of the conclusions. We commend the authors for their efforts and hope that our comments will promote further methodological refinements in genetic association studies of autoimmune diseases.

## Data Availability

Data sharing is not applicable to this article as no data were created or analysed in this research.

## Open scholarship

This article has earned the Center for Open Science badges for Open Data, Open Materials and Preregistered. The data and materials are openly accessible at re3data.org.
